# Supplementary material for: Intergenerational trauma transmission is associated with brain metabotranscriptome remodeling and mitochondrial dysfunction
Source: Commun Biol. 2021 Jun 24;4:783. doi: 10.1038/s42003-021-02255-2 (PMC8225861; doi:10.1038/s42003-021-02255-2)

**Supplementary Information**

# Intergenerational Trauma Transmission is Associated with Brain Metabotranscriptome Remodeling and Mitochondrial Dysfunction

Sammy Alhassen[1a], Siwei Chen[2,3a], Lamees Alhassen[1], Alvin Phan[1], Mohammad Khoudari[1], Angele De Silva[4], Huda Barhoosh[1], Zitong Wang[1], Chelsea Parrocha[1], Emily Shapiro[1], Charity Henrich[1], Zicheng Wang[1], Leon Mutesa[5], Pierre baldi[2,3], Geoffrey Abbott[4], Amal Alachkar[1,3*]

1 Department of Pharmaceutical Sciences, University of California-Irvine, CA 92697

2 Department of Computer Science, School of Information and Computer Sciences, University of California-Irvine, CA 92697

3 Institute for Genomics and Bioinformatics, School of Information and Computer Sciences, University of California-Irvine, CA 92697

4 Bioelectricity Laboratory, Department of Physiology and Biophysics, School of Medicine, University of California-Irvine, CA 92697

5 Center for Human Genetics, College of Medicine and Health Sciences, University of Rwanda, Kigali

[a] These authors contributed equally to this work

***Corresponding Authors**

Dr. Amal Alachkar

Department of Pharmaceutical Sciences

University of California, Irvine, CA 92697

aalachka@uci.edu

## Supplementary Figures

**Supplementary Figure 1. Prenatal exposure to stress produces impairments in maternal behavior and depressive-like behavior in mothers**

**a-d.** Exposure to stress produces maternal behavior deficits

**a,b.** Pups retrieval latency and duration: **(a)** Time to retrieve one pup (n=13 c→C, 12 s→S), Mann Whitney test (U=24.50, *P*=0.0025): c→C vs s→S, ** *P*<0.01. (**b)** Time to retrieve all pups (n=13 c→C, 12 s→S). Mann Whitney test (U=19.50, *P*=0.0008): c→C vs s→S, *** *P*<0.001.

**c.** Time mice spent immobile in the forced swim assay (n=10 c→C, 10 s→S). Unpaired student test (t=6.815, *P*<0.0001): c→C vs s→S, *** *P*<0.001.

**d-g**. Mother behaviors in postpartum day 13 (PPD13) in the locomotor activity box. Total **(d)** distance travelled**, (e)** vertical counts, and **(f)** stereotypic behavior, measured in 60 minutes, unpaired t-test, *P* > 0.05 for all tests.

**g.** Time spent in the central and peripheral zones by mothers on PPD13, Two way ANOVA, *P* > 0.05.

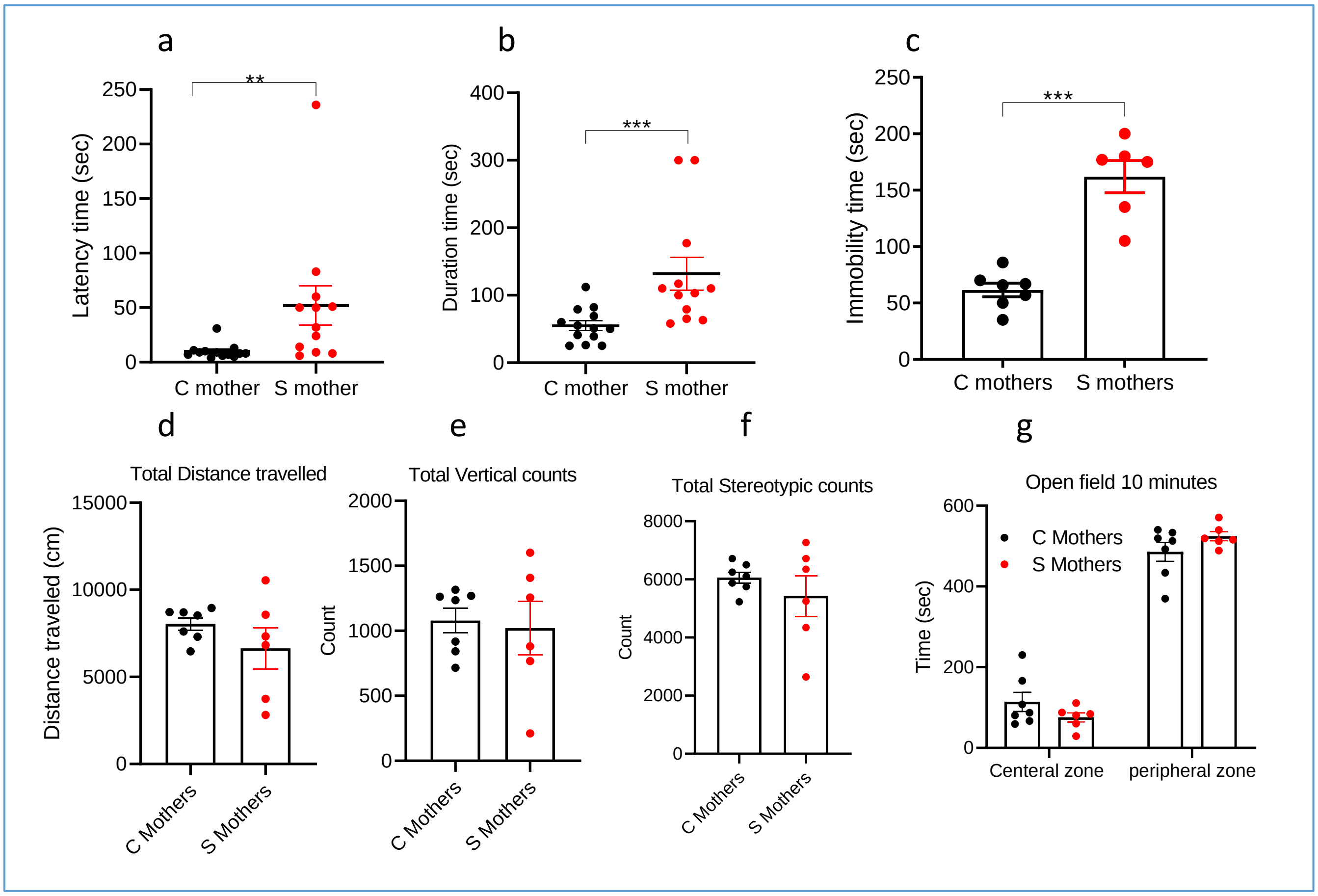

**Supplementary Figure 2. Bioinformatics analysis of the pathways enriched in DEGs in the brains of neonatal s→S mice**

**a-f:** three top differentially regulated TFs [Fos **(a,b),** Egr1 **(c,d),** and Sp4 **(e,f)**] in the 24-hr mice with their differential target genes identified using **(a,c,e)** MotifMap and **(b,d,f)** ChiPseq (Blue: downregulated genes; Red: upregulated genes).

**g,h.** network of differentially regulated TFs **(n)** and RBPs **(o)** in the 24-hr mice, with their differential target genes identified by MotifMap.

Supplementary Data 9 and 10 show detailed lists of the genes present in networks.

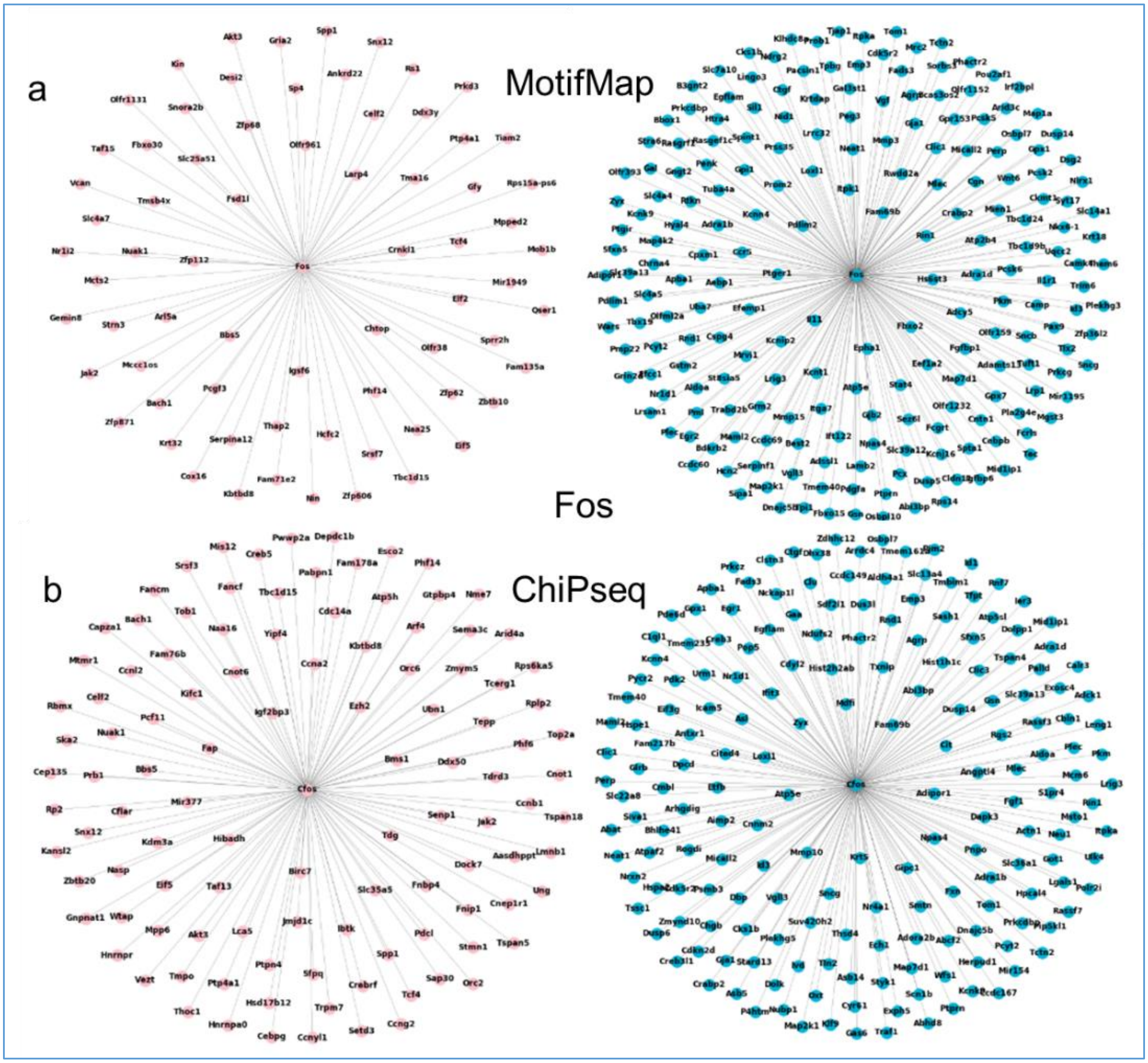

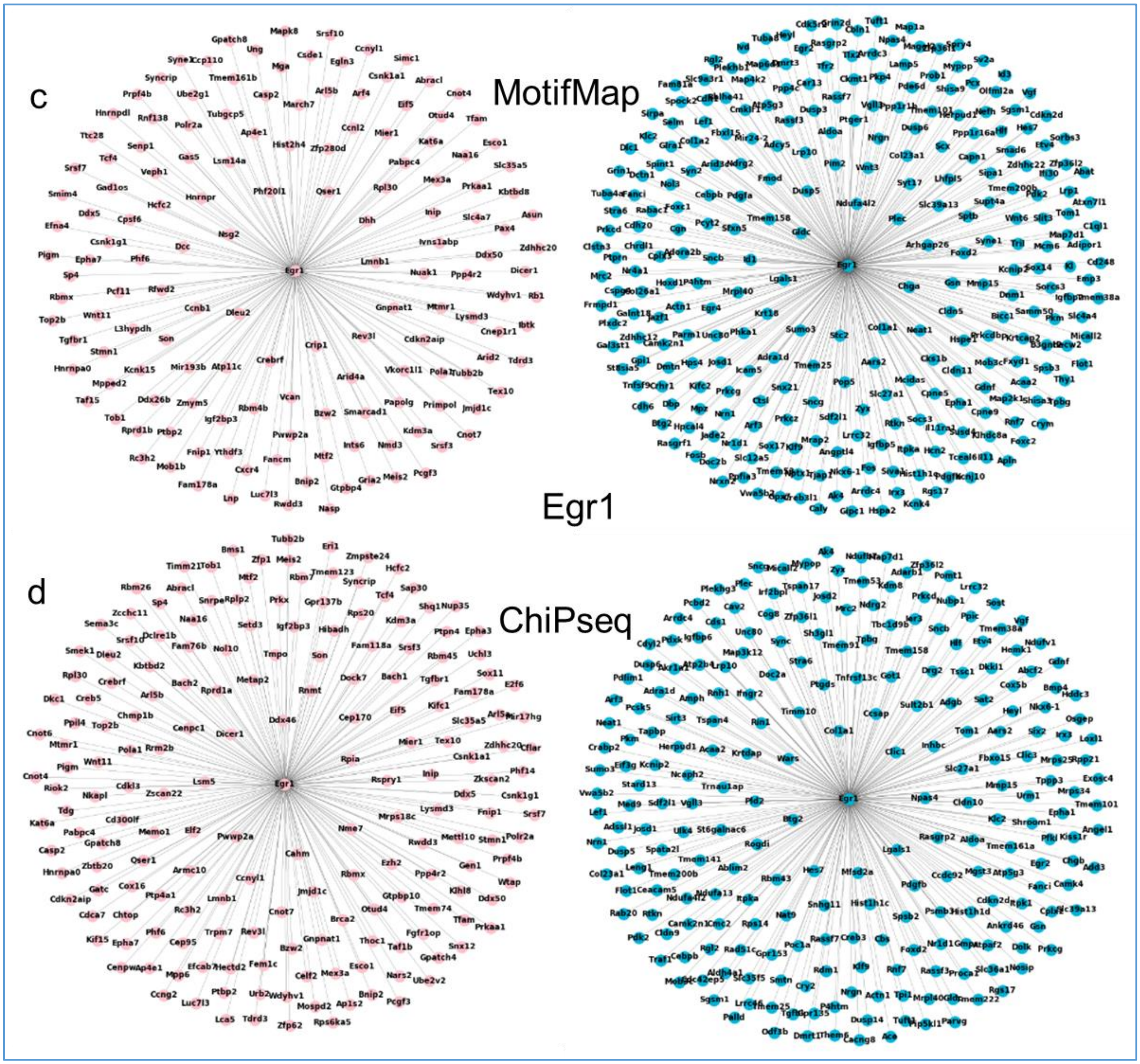
c
MotifMap
Egr1
d
ChiPseq

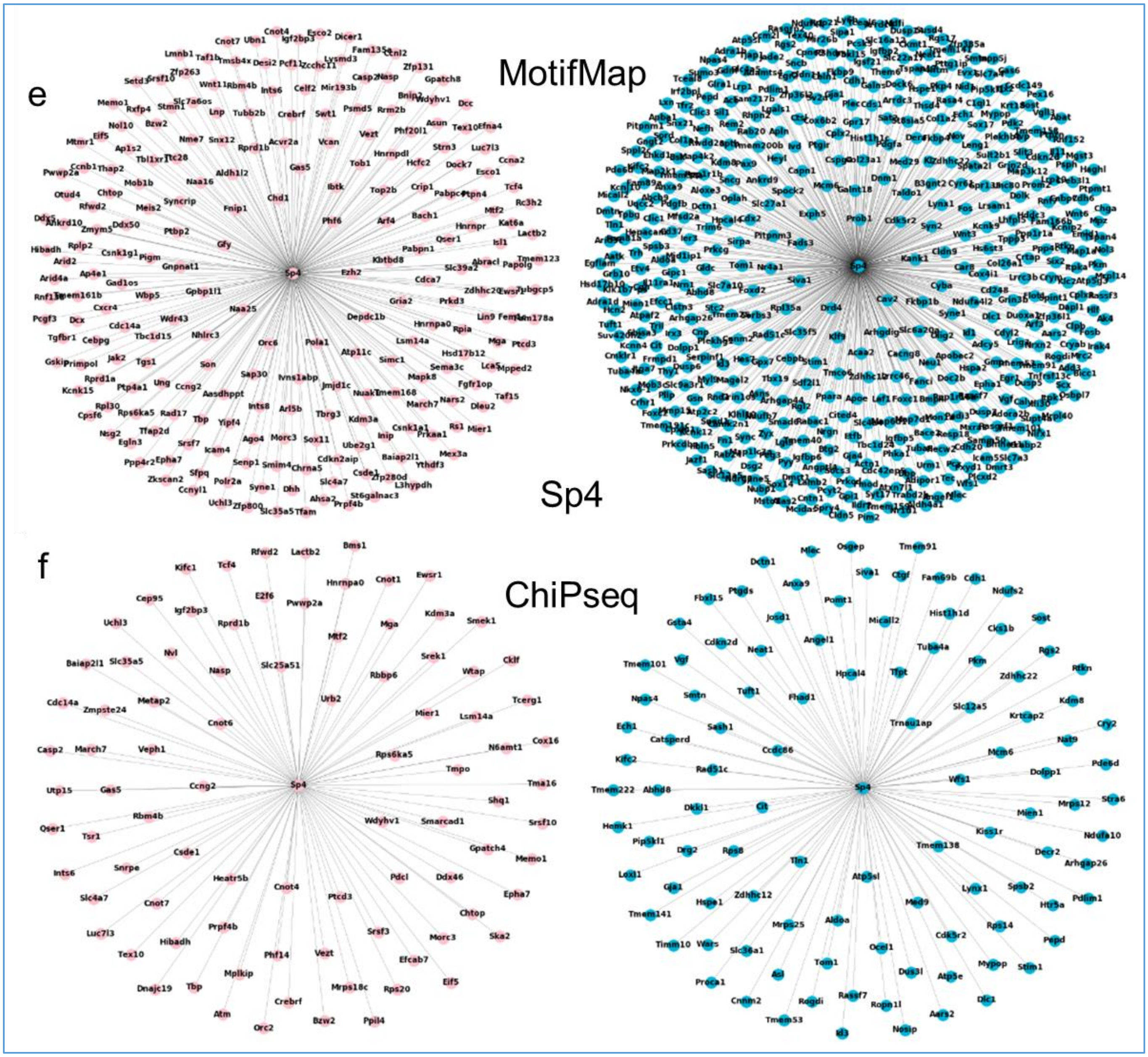
e
MotifMap
Sp4
f
ChiPseq

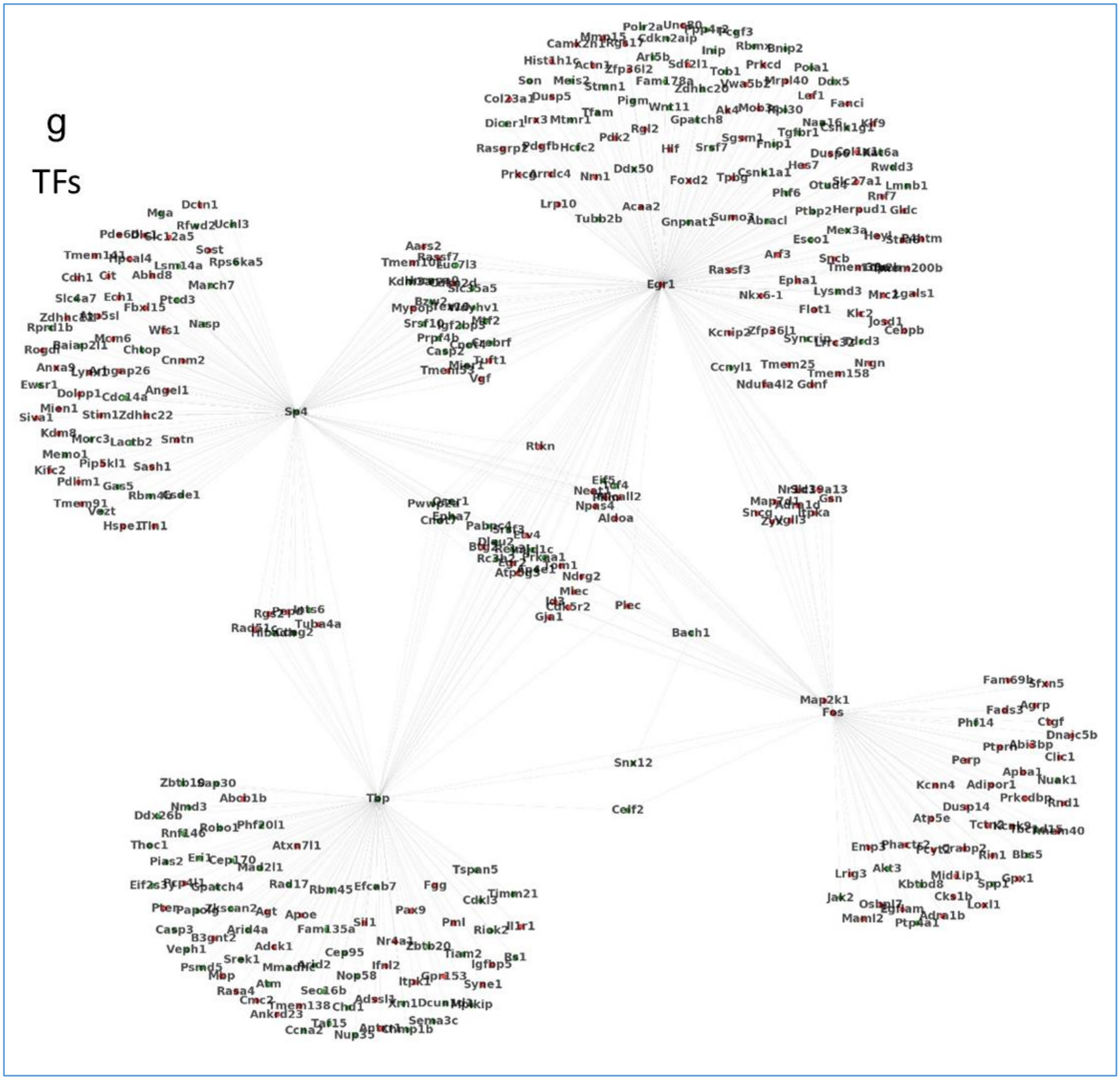
g
TFs
Egr1
Sp4
Tbp
Map2k1
Fos
Rtkn
Snx12
Celf2
Bach1
Plec
Aldoa
Ndrg2
Gja1
Rassf3
Nkx6-1
Lrp10
Tubb2b
Acaa2
Gnpnat1
Ddx50
Foxd2
Hes7
Phf6
Fancl
Lef1
Ddx5
Rwdd3
Lmnb1
Gldc
Pola1
Bnip2
Inip
Tfam
Wnt11
Gpatch8
Pigm
Pdk2
Hlf
Srsf7
Nrn1
Ccnyl1
Tmem25
Tmem158
Nrgn
Gdnf
Josd1
Cebpb
Epha1
Flot1
Klc2
Arf3
Sncb
Eseo1
Mex3a
Kcnip2
Sost
Rps6ka5
March7
Pted3
Nasp
Wfs1
Mcm6
Chtop
Cnnm2
Angel1
Stim1
Zdhhc22
Smtn
Sash1
Gas5
Pdlim1
Tmem91
Vezt
Kifc2
Memo1
Siva1
Ewsr1
Anxa9
Rogdi
Rprd1b
Slc4a7
Cdh1
Mga
Dctn1
Uchl3
Rfwd2
Aars2
Slc35a5
Srsf10
Prpf4b
Casp2
Tmem55
Vgf
Tuft1
Npas4
Pwwp2a
Epha7
Etv4
Prkaa1
Rc3h2
Rgs2
Tuba4a
Rad51c
Nrsn1
Slc30a13
Gsn
Sncg
Zyx
Itpka
Fam69b
Sfxn5
Agrp
Fads3
Phf14
Ctgf
Dnajc5b
Abi3bp
Clic1
Perp
Apba1
Kcnn4
Adipor1
Nuak1
Prkcdbp
Rnd1
Dusp14
Atp5e
Tmem40
Emp3
Rin1
Bbs5
Lrig3
Akt3
Kbtbd8
Gpx1
Spp1
Jak2
Cks1b
Loxl1
Maml2
Adra1b
Zbtb10
Sap30
Abcb1b
Ddx26b
Nmd3
Robo1
Phf20l1
Rnf146
Thoc1
Atxn7l1
Pias2
Eri1
Cep170
Mad2l1
Eif2s3y
Gpatch4
Rad17
Rbm45
Efcab7
Tspan5
Fgg
Timm21
Cdkl3
Pten
Agt
Apoe
Pax9
Casp3
Arid4a
Fam135a
Sil1
Pml
Riok2
Il1r1
B3gnt2
Veph1
Adck1
Cep95
Nr4a1
Zbtb20
Tiam2
Srek1
Ifnl2
Igfbp5
Psmd5
Mbp
Mmadhc
Arid2
Nop58
Gpr153
Atm
Itpk1
Syne1
Rasa4
Sec16b
Chd1
Cmc2
Tmem138
Adssl1
Xrn1
Mpkip
Ankrd23
Taf15
Sema3c
Ccna2
Nup35
Chmp1b

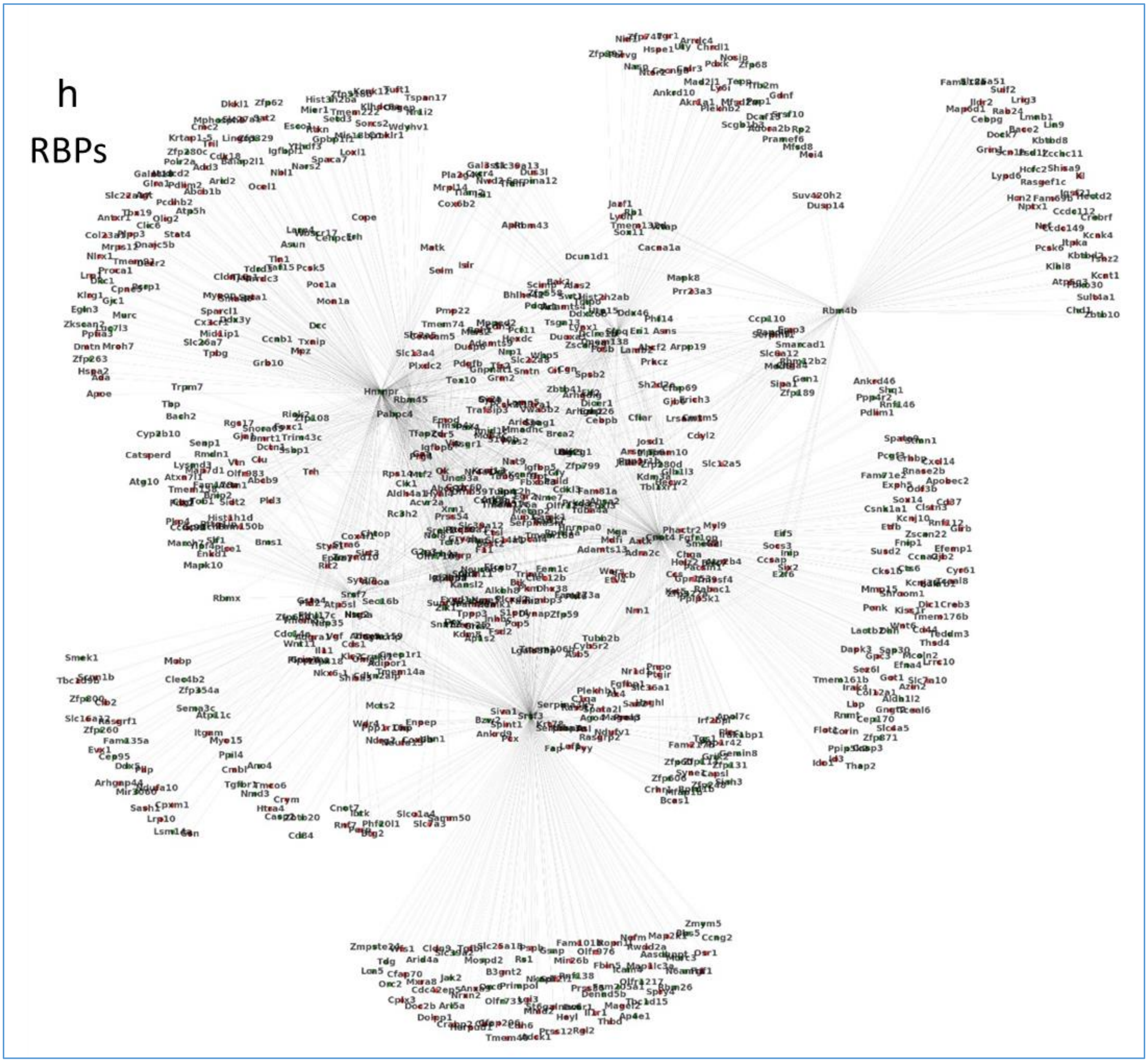
h
RBPs

**Supplementary Figure 3. Bioinformatics analysis of the pathways enriched in DEGs in the brains of adult s→S mice**

**a-f:** three top differentially regulated TFs [Fos **(a,b),** Egr1 **(c,d),** and Sp4 **(e,f)**] in the adult mice with their differential target genes identified using **(a,c,e)** MotifMap and **(b,d,f)** ChiPseq (Blue: downregulated genes; Red: upregulated genes)

**g,h.** network of differentially regulated TFs **(g)** and RBPs **(h)** in the adult mice, with their differential target genes identified by MotifMap.

Supplementary Data 9 and 10 show detailed lists of the genes present in networks.

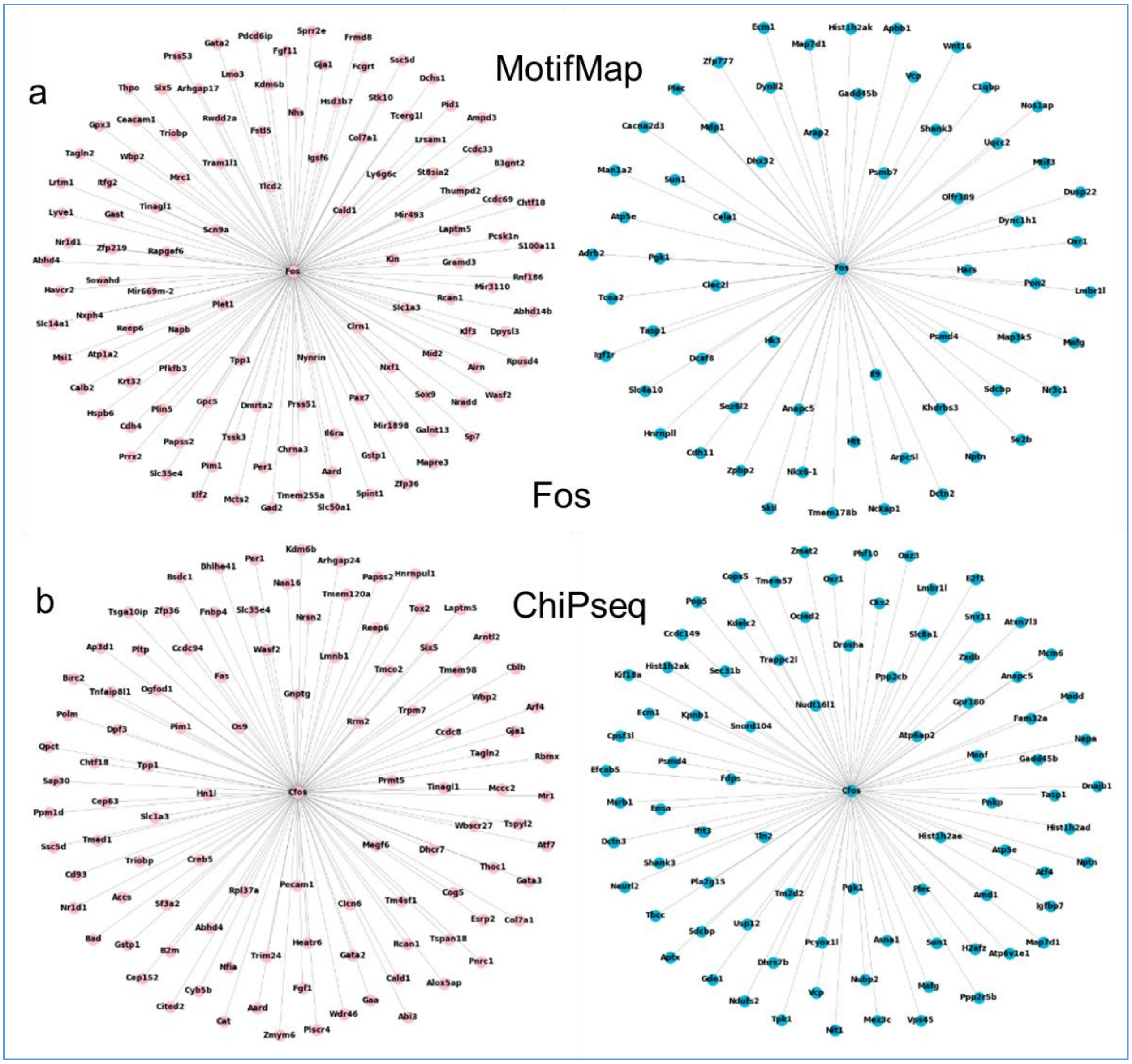
a
MotifMap
Fos
b
ChiPseq

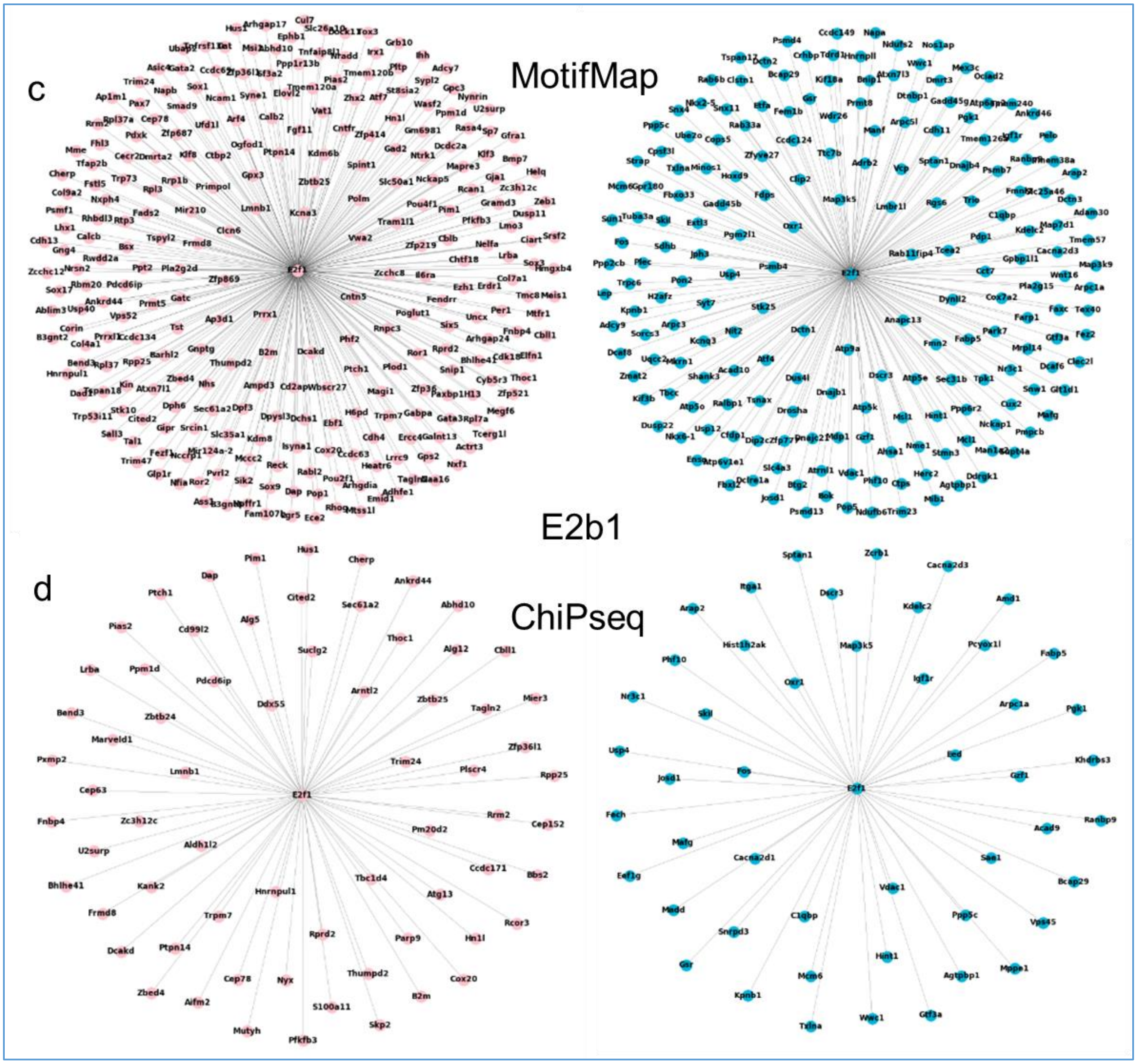
c
MotifMap
E2f1
E2b1
d
ChiPseq
E2f1

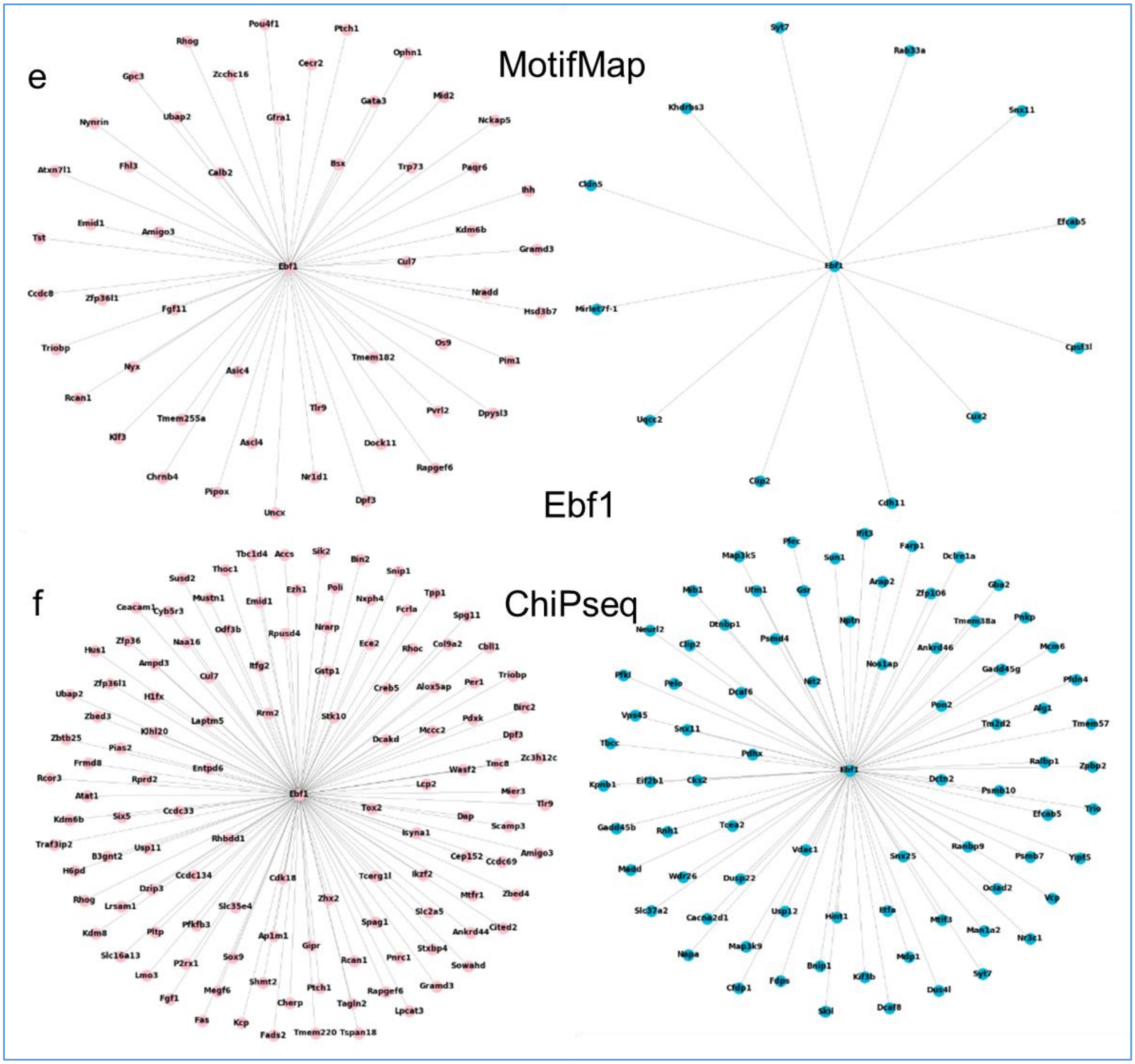
e
MotifMap
Ebf1
f
ChiPseq
Ebf1
Pou4f1
Ptch1
Rhog
Ophn1
Cecr2
Zcchc16
Gpc3
Gata3
Mid2
Ubap2
Gfra1
Nynrin
Nckap5
Bsx
Trp73
Paqr6
Fhl3
Calb2
Atxn7l1
Ihh
Emid1
Kdm6b
Amigo3
Tst
Gramd3
Cul7
Nradd
Ccdc8
Zfp36l1
Fgf11
Hsd3b7
Os9
Triobp
Tmem182
Pim1
Nyx
Asic4
Rcan1
Tlr9
Pvrl2
Dpysl3
Tmem255a
Klf3
Ascl4
Dock11
Rapgef6
Chrnb4
Nr1d1
Pipox
Dpf3
Uncx
Syt7
Rab33a
Khdrbs3
Snx11
Cldn5
Efcab5
Mirlet7f-1
Cpsf3l
Uqcc2
Cux2
Clip2
Cdh11
Tbc1d4
Accs
Sik2
Bin2
Snip1
Susd2
Thoc1
Ezh1
Poli
Tpp1
Mustn1
Emid1
Nxph4
Fcrla
Ceacam1
Cyb5r3
Spg11
Odf3b
Rpusd4
Nrarp
Zfp36
Naa16
Ece2
Rhoc
Col9a2
Cbll1
Hus1
Ampd3
Rtfg2
Gstp1
Cul7
Zfp36l1
Triobp
H1fx
Creb5
Alox5ap
Per1
Ubap2
Rrm2
Laptm5
Stk10
Pdxk
Birc2
Zbed3
Klhl20
Mccc2
Dcakd
Dpf3
Zbtb25
Pias2
Tmc8
Zc3h12c
Frmd8
Entpd6
Wasf2
Rcor3
Rprd2
Lcp2
Mier3
Atat1
Tox2
Tlr9
Ccdc33
Dap
Kdm6b
Six5
Scamp3
Isyna1
Traf3ip2
Rhbdd1
Usp11
Cep152
Ccdc69
Amigo3
B3gnt2
H6pd
Tcerg1l
Ikzf2
Ccdc134
Cdk18
Mtfr1
Zbed4
Dzip3
Zhx2
Rhog
Lrsam1
Slc35e4
Slc2a5
Spag1
Cited2
Kdm8
Pltp
Pfkfb3
Ankrd44
Ap1m1
Slc16a13
Gipr
Stxbp4
Sox9
Pnrc1
P2rx1
Rcan1
Sowahd
Lmo3
Shmt2
Ptch1
Rapgef6
Gramd3
Megf6
Fgf1
Cherp
Tagln2
Lpcat3
Fas
Kcp
Fads2
Tmem220
Tspan18
Ifit3
Pfec
Farp1
Map3k5
Son1
Dclre1a
Mib1
Ufm1
Arap2
Gba2
Gsr
Zfp106
Neurl2
Dtnbp1
Nptn
Tmem38a
Pnkp
Clip2
Psmd4
Ankrd46
Mcm6
Pfkl
Nos1ap
Gadd45g
Pelo
Dcaf6
Pfdn4
Vps45
Pon2
Afg1
Snx11
Tmem57
Tbcc
Pdhx
Ralbp1
Zpbp2
Kpnb1
Eif2b1
Cks2
Dctn2
Psmb10
Trio
Efcab5
Gadd45b
Rab1
Tcea2
Ranbp9
Psmb7
Vdac1
Snx25
Ypf5
Wdr26
Dusp22
Ociad2
Vcp
Slc37a2
Cacna2d1
Usp12
Hint1
Etfa
Man1a2
Nr3c1
Napa
Map3k9
Mdp1
Bnip1
Kif3b
Dus4l
Syt7
Cfdp1
Fdps
Skil
Dcaf8

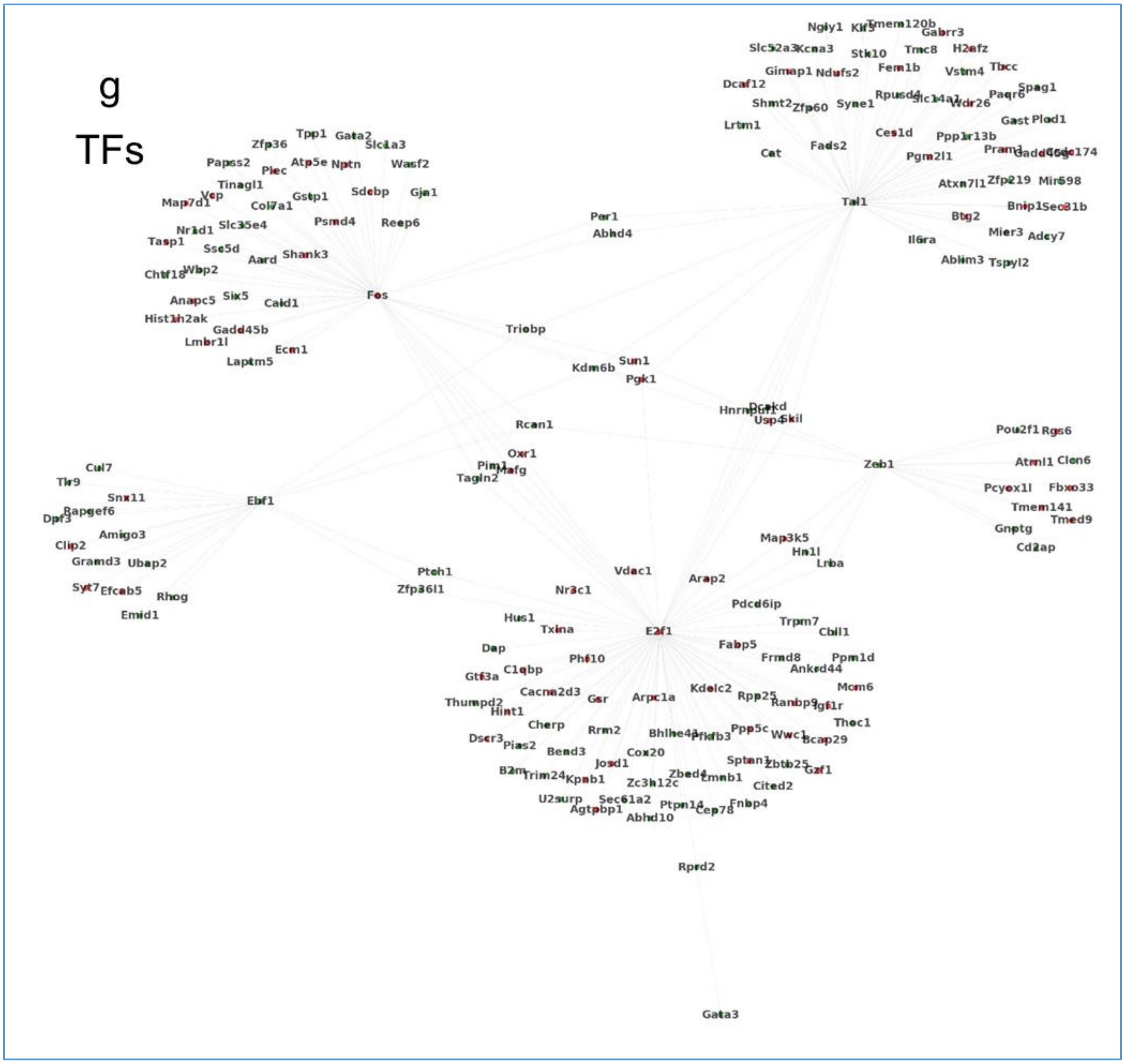
g
TFs
Fos
Tal1
Ebf1
Zeb1
E2f1
Per1
Abhd4
Triobp
Sun1
Kdm6b
Pgk1
Rcan1
Oxr1
Pim1
Mafg
Tagln2
Skil
Usp4
Vdac1
Nr3c1
Ptch1
Zfp36l1
Rprd2
Gata3
Tpp1
Gata2
Slc1a3
Zfp36
Papss2
Plec
Atp5e
Nptn
Wasf2
Tinagl1
Vcp
Map7d1
Col7a1
Gstp1
Sdcbp
Gja1
Nr1d1
Slc35e4
Psmd4
Reep6
Tasp1
Sssc5d
Aard
Shank3
Wbp2
Anapc5
Six5
Cald1
Gadd45b
Lmbr1l
Ecm1
Laptm5
Ngly1
Klf5
Tmem120b
Gabrr3
Slc52a3
Kcna3
Stk10
Tmc8
H2afz
Gimap1
Ndufs2
Fem1b
Vstm4
Tbcc
Dcaf12
Spag1
Shmt2
Zfp60
Syne1
Paqr6
Wdr26
Gast
Plod1
Lrtm1
Ces1d
Ppp1r13b
Cat
Fads2
Pgm2l1
Pram1
Atxn7l1
Zfp219
Mir598
Bnip1
Sec31b
Btg2
Mier3
Adcy7
Il6ra
Ablim3
Tspyl2
Cul7
Tlr9
Snx11
Rapgef6
Dpf3
Amigo3
Clip2
Gramd3
Ubap2
Syt7
Efcab5
Rhog
Emid1
Pou2f1
Rgs6
Atml1
Cln6
Pcyox1l
Fbxo33
Tmem141
Tmed9
Gnptg
Cd2ap
Map3k5
Lrba
Arap2
Pdcd6ip
Trpm7
Cbll1
Hus1
Txina
Dap
Phf10
Fabp5
Frmd8
Ppm1d
Ankrd44
C1qbp
Gtf3a
Cacna2d3
Gsr
Arpc1a
Kdelc2
Rpp25
Mcm6
Thumpd2
Hint1
Ranbp9
Igf1r
Cherp
Rrm2
Ppp5c
Thoc1
Dscr3
Pias2
Bend3
Cox20
Pfkfb3
Wwc1
Bcap29
Josd1
Sptan1
Zbtb25
Gzf1
B2m
Trim24
Kpnb1
Zc3h12c
Zbed4
Lmnb1
Cited2
U2surp
Sec61a2
Ptpn14
Cep78
Fnbp4
Agtpbp1
Abhd10

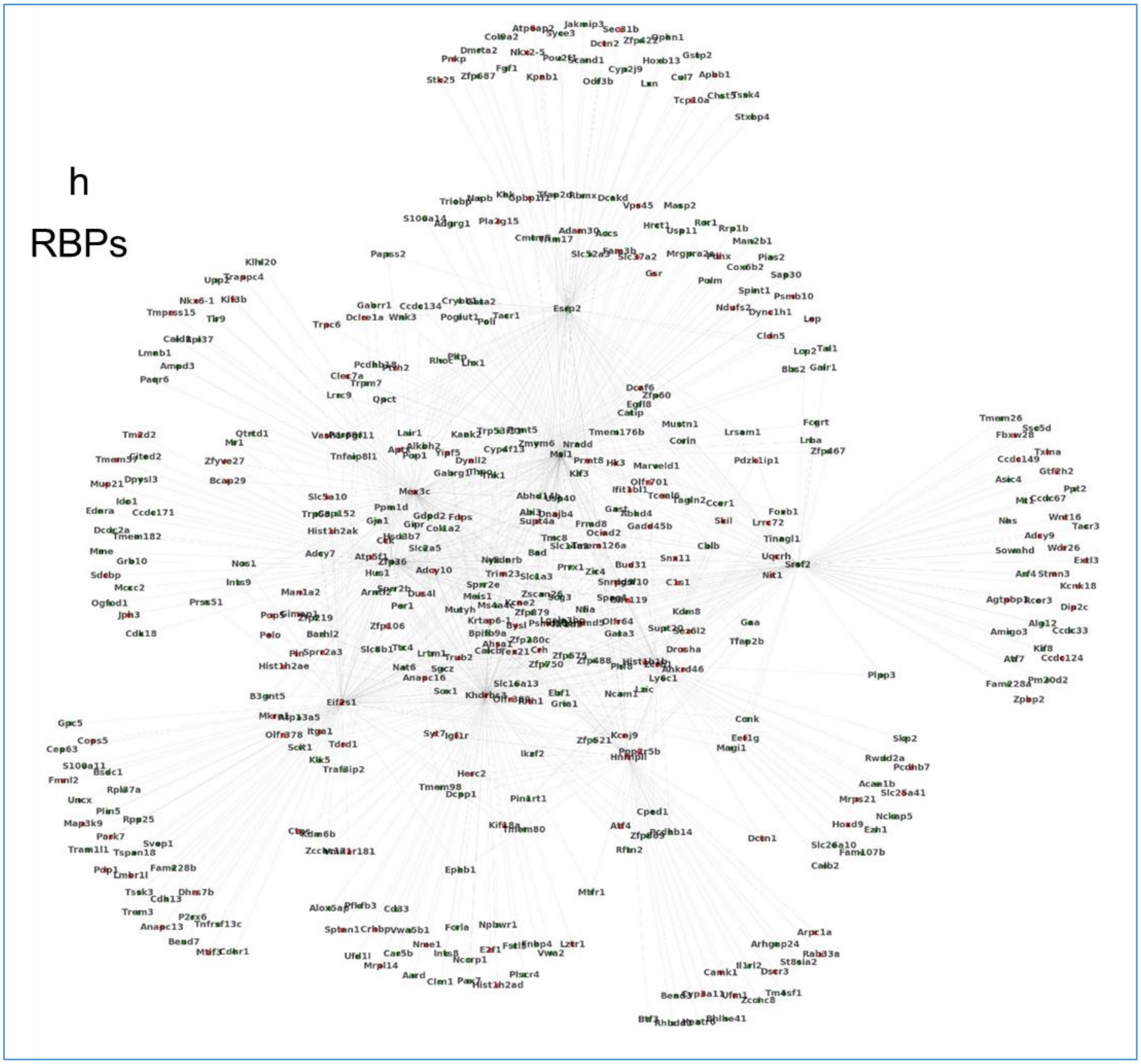
h
RBPs

**Supplementary Figure 4. Immunohistochemistry analysis of Arc in the brains of adult s→S and c→C mice.**

**a,c,e,g,** Representative images of Arc-immunoreactivity in the **(a)** PFC, **(c)** Hip, **(e)** NAc, **(g)** Str

**b,d,f,h,** quantification of Arc immuno-positive cells in the **(b)** PFC, **(d)** Hip, **(f)** NAc, (**h)** Str

Unpaired t-test, PFC: t = 0.198, *P* = 0.85, n = 4; Hip: t=6.678, *P* = 0.001, n = 3 c→C 4 s→S; Nac: t=0.26, *P* = 0.8, n = 4; Str: t=2.87, *P* = 0.028, n = 4; Scale bar = 20uM. Values represent mean ± SEM. Frontal cortex (PFC), hippocampus (Hip), nucleus accumbens (NAc), striatum (Str). Scale bar 50 µm. Data are presented as means±S.E.M

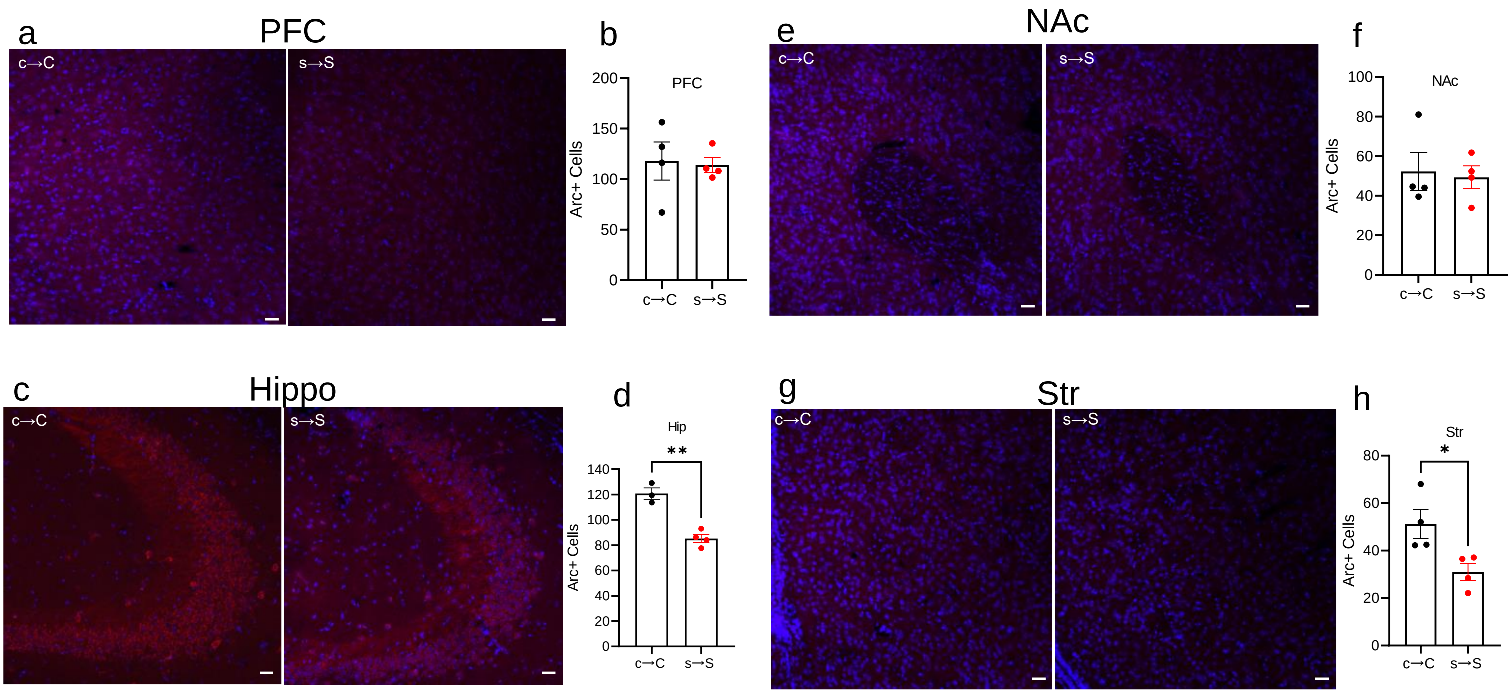

Supplement: Supplementary file 1 — Supplementary Information [file 42003_2021_2255_MOESM1_ESM.pdf]
