# Supplementary material for: Intergenerational trauma transmission is associated with brain metabotranscriptome remodeling and mitochondrial dysfunction
Source: Commun Biol. 2021 Jun 24;4:783. doi: 10.1038/s42003-021-02255-2 (PMC8225861; doi:10.1038/s42003-021-02255-2)
Supplement: Supplementary file 2 — Description of Additional Supplementary Files [file 42003_2021_2255_MOESM2_ESM.pdf]

Description of Additional Supplementary Files

File name: Supplementary Data 1

Description: Metabolites' differential expressions in the brains of neonatal and adult mice.

File name: Supplementary Data 2

Description: Analysis of enrichment pathways in the differential metabolites.

File name: Supplementary Data 3

Description: Gene differential expressions in the brains of neonatal and adult mice.

File name: Supplementary Data 4

Description: Hypergeometric analysis of enrichment pathways in the differential genes.

File name: Supplementary Data 5

Description: Differential TFs and RBPs and their differential targets.

File name: Supplementary Data 6

Description: Metabolomic-transcriptomic integration analysis.

File name: Supplementary Data 7

Description: Integrative analysis of differential genes in mice Human MDD.

File name: Supplementary Data 8

Description: Source data underlying the graphs.

File name: Supplementary Data 9

Description: ChIPseq analysis of TFs targets in the neonatal and adult pups.

File name: Supplementary Data 10

Description: MotifMap analysis of TFs targets in the neonatal and adult pups.
